# A dPCR Method for Quantitative Authentication of Wild Lingonberry (*Vaccinium vitis-idaea*) versus Cultivated American Cranberry (*V. macrocarpon*)

**DOI:** 10.3390/foods11101476

**Published:** 2022-05-19

**Authors:** Katja Karppinen, Anna Avetisyan, Anne Linn Hykkerud, Laura Jaakola

**Affiliations:** 1 Department of Arctic and Marine Biology, UiT the Arctic University of Norway, NO-9037 Tromsø, Norway; katja.karppinen@uit.no (K.K.); anna.avetisyan@uit.no (A.A.); 2 NIBIO, Norwegian Institute of Bioeconomy Research, Department of Horticulture, NO-1431 Ås, Norway; anne.linn.hykkerud@nibio.no

**Keywords:** *Vaccinium* berries, chloroplast genome, digital PCR, authentication, quantification, DNA barcoding

## Abstract

Berries of the genus *Vaccinium* are highly valued health-beneficial superfoods, which are commonly subjected to adulteration and mixed with each other, or with other common berry species. A quantitative DNA-based method utilizing a chip-based digital polymerase chain reaction (dPCR) technique was developed for identifying and quantifying wild lingonberry (*V. vitis-idaea*) and cultivated American cranberry (*V. macrocarpon*). The dPCR method with species-specific primers for mini-barcoding was designed based on the indel regions found in the *trnI-CAU–trnL-CAA* locus in the chloroplast genome. The designed primers were able to amplify only target species, enabling to distinguish the two closely related species with good sensitivity. Our results illustrated the ability of the method to identify lingonberry and American cranberry DNA using PCR without the need for probes or further sequencing. The dPCR method could also quantify the DNA copy number in mixed samples. Based on this study, the method provides a basis for a simple, fast, and sensitive quantitative authentication analysis of lingonberry and American cranberry by dPCR. Moreover, it can also provide a platform for authentication analyses of other plant species as well by utilizing the indel regions of chloroplast genomes.

## 1. Introduction

**Figure 1:**
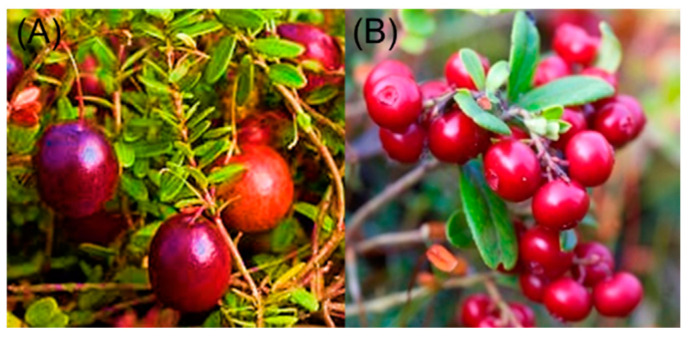
Cultivated American cranberry (**A**) and wild lingonberry (**B**).

The *Vaccinium* genus includes a number of commercially important berry-producing species, which are recognized for their health-beneficial attributes and are considered worldwide as “superfoods”. Some of the most important species are cultivated and semi-cultivated blueberries (*V. corymbosum*, *V. angustifolium*, *V. ashei*) and American cranberries (*V. macrocarpon*), in addition to bilberries (*V. myrtillus*) and lingonberries (*V. vitis-idaea*), which are mostly utilized as a wild crop [1]. Cranberries and lingonberries share similarities in their red color (Figure 1) as well as astringent taste, which are both affected by the abundance of phenolic compounds [2,3]. In addition to these similarities, they share closely related genomes [4,5]. However, wild lingonberries generally contain higher level of phenolic compounds [2,3] and are more valued in the world market than cultivated cranberries and can therefore potentially be substituted by cranberries in products. Mislabeled and fraudulent lingonberry products with partial or total replacement with American cranberries have been reported [6,7]. Food fraud is often committed with the deliberate intention to gain economic benefit and to mislead customers, who are increasingly more health aware. Even if fraud would not cause immediate health risks, they may reduce customer trust in food suppliers and should be able to be detected. For this reason, reliable and sensitive methods for the authentication of these closely related *Vaccinium* species are needed.

Chemical analysis methods are widely used for the authentication of plant-based food products, and various methods have been optimized specifically for berries [8]. Anthocyanin profiles have been used to distinguish lingonberries and American cranberries in dietary supplement products [6]. Hurkova et al. (2019) [7] characterized the phytochemical diversity of these species via non-targeted metabolomic fingerprinting for identifying metabolites that could be used as selective markers between lingonberries and cranberries. The representatives of polyphenols and phospholipids were found to contribute as markers in the classification of these berries that have fairly similar anthocyanin profiles, but could be diversified based on peonidin-3-O-arabinoside and myricetin 3-O-glucoside, which were present in the cranberry flavonoid profile, but not detected in lingonberries. Recently, the UPLC-PDA method was developed, based on the presence of mainly 3-galactosides of cyanidin and peonidin of anthocyanins in cranberry versus cyanidin and its glycosides with just traces of other anthocyanins in lingonberry [9].

In some cases, metabolite profiles are influenced by external environmental factors, such as light, temperature, or storage conditions. For this reason, DNA-based authentication methods are of interest when applicable. DNA barcoding is widely applied for molecular identification in studies of the taxonomical relationships of species, population genetics, in trade control of illegal wildlife collection, and in monitoring food and medical product authenticity and fraud [10]. However, for authentication between berry species, relatively few DNA-based methods have been developed [8]. For small berry species, DNA barcoding technology based on Sanger sequencing [11] and high-resolution melting (Bar-HRM) have been developed [12]. The latter method provides a relatively rapid high-throughput analysis for qualitative diversification between different species, but it is not suitable for the quantification of fraud in berry mixes.

Many of the utilized DNA barcoding methods for authentication are qualitative, meaning that they are able to show fraud in products, but not quantify the level of authentic versus fraud raw materials. Digital PCR (dPCR) is a powerful technique for the absolute quantification of the DNA copy number, with a system based on either droplets (ddPCR) or chips [13]. Compared to quantitative PCR (qPCR), dPCR is more sensitive and does not require calibration or internal controls. dPCR techniques have been widely used in clinical diagnostics but also in the authentication of species and food products, especially meat and genetically modified crops [14,15,16,17]. The authentication of plant-based products using dPCR has been successful with, for instance, olive oil [18] and *Panax* herbs [19].

In this study, we developed a fast and efficient dPCR method for the authentication of lingonberry and American cranberry utilizing the earlier published complete chloroplast genomes of these species [20]. The detected indel regions enabled the designing of species-specific primers with amplicon lengths of less than 200 bp, which are suitable for mini-barcoding that allows amplification from partly degraded DNA from processed food products. The optimized method could not only discriminate between lingonberry and American cranberry DNA but was also able to quantify the DNA copy numbers in mixed samples.

## 2. Materials and Methods

### 2.1. Plant Material and DNA Extraction

Wild lingonberry (*Vaccinium vitis-idaea*) leaf and berry material originated from a natural forest stand in Tromsø, Norway. American cranberry (*Vaccinium macrocarpon*) material in the form of powdered berries was received from Linards Klavins, University of Latvia. Prior to DNA extraction, the plant material was grounded to fine powder under liquid nitrogen using a mortar and pestle. DNA was extracted from 50 mg of plant powder using an E.Z.N.A. HP Plant DNA Mini Kit (Omega Bio-tek, Norcross, GA, USA). The DNA was qualified and quantified using a NanoDrop^TM^ 2000c spectrophotometer (Thermo Fischer Scientific, Foster City, CA, USA).

### 2.2. Primer Design and Specificity

Publicly available complete chloroplast genome sequences of lingonberry (GenBank accession no. LC52969) and American cranberry (GeneBank accession no. NC_019616) were aligned and compared using Clustal Omega (https://www.ebi.ac.uk/Tools/msa/clustalo/, accessed on 1 January 2021). Primers were designed from variable sequence regions using the Primer3 program (https://bioinfo.ut.ee/primer3-0.4.0/, accessed on 1 January 2021) with amplicon sizes of less than 200 bp. Primer specificity to target species DNA was tested using conventional PCR in a 25 μL reaction volume containing 2.5 μL of 10× DreamTaq Buffer (20 mM MgCl_2_), 0.5 μL of dNTP mixture (each 10 mM), 0.125 μL of DreamTaq DNA Polymerase (5 U/μL; Thermo Fischer Scientific), 10 ng of lingonberry or cranberry DNA, and 1 μL of forward and reverse primers (5 μM). The PCR conditions were an initial incubation at 94 °C for 5 min followed by 32 cycles at 94 °C for 1 min, 63 °C for 1 min, and 72 °C for 1 min, with a final extension at 72 °C for 10 min. PCR products were visualized in 1% agarose gel with ethidium bromide.

The designed primers were further tested for specificity and efficiency by qPCR using an MJ MiniOpticon instrument (Bio-Rad, Hercules, CA, USA) and CFX Manager software 2.0 (Bio-Rad). The 15 μL reaction volume included 7.5 μL of SsoFast™ EvaGreen Supermix (Bio-Rad), 10 ng of lingonberry or cranberry DNA template, and 1.5 μL of forward and reverse primers (5 μM). The qPCR conditions included an initial incubation at 96 °C for 10 min followed by 40 cycles of 98 °C for 30 s, and 63 °C for 2 min, followed by a melting curve analysis (ranging from 65 °C to 95 °C with increment of 0.5 °C every cycle), for the validation of the amplification of only one product. The qPCR products were sequenced to verify the target sequence by using a BigDye Terminator Cycle Sequencing Kit (Applied Biosystems, Foster City, CA, USA) and the sequencing was performed at the UiT The Arctic University of Norway sequencing facility on a 3130xl Genetic Analyzer (Applied Biosystems). Primer efficiency was validated by qPCR using calibration curves consisting of five 10-fold dilutions of target DNA and calculated using the following equation: Efficiency (%) E = (10^^(−1/slope)−1^) × 100. Based on the results from PCR and qPCR, one primer pair for both species was selected for further analysis in dPCR (Table 1).

### 2.3. Quantitative Chip-Based Digital-PCR (dPCR) with DNA Mixes

The optimal DNA amount/copy number for dPCR was initially tested using 1.5, 3.0, 7.5 and 15 ng DNA per reaction to ensure that the target DNA copy number in the reaction well of the chip fell within the digital range suggested by the manufacturer. The amount of 7.5 ng sample showed a copy number under 2000 copies/μL and was further utilized for dPCR experiments. DNA samples extracted from lingonberry and cranberry fruit were mixed in the ratios of 0%, 1%, 5%, 10%, 25%, 50%, 75%, 90%, 95%, 99%, and 100% (*w*/*w*). For the DNA copy number analysis, SYBR^®^ Green I (Thermo Fisher Scientific, Waltham, MA, USA) on the QuantStudio^TM^ 3D Digital PCR system (Applied Biosystems by Thermo Fisher Scientific, Waltham, MA, USA) with FAM detection channel was used. The reaction volume was 15 μL containing 7.5 μL of 2× QuantStudio^TM^ 3D Digital PCR Master Mix, 0.6 μL of each primer (5 mM), 1.5 μL SYBR Green I^®^ (2% DMSO), 1.8 μL nuclease-free water and 3 μL DNA template (7.5 ng). The reaction mixture was immediately loaded onto chips using a QuantStudio^TM^ 3D Digital PCR Chip Loader (Thermo Fisher Scientific, Waltham, MA, USA). PCR amplification was performed in a GeneAmp PCR System 9700 thermal cycler (Applied Biosystems) with the following thermal parameters: 96 °C for 10 min followed by 40 cycles at 63 °C for 2 min and at 98 °C for 30 s, and a final extension at 63 °C for 2 min. Data analysis was carried out using the QuantStudio^TM^ 3D AnalysisSuite Cloud Software v3.1 (Thermo Fisher Scientific, Waltham, MA, USA). The dPCR analysis was conducted with at least three replicates for each mixture.

## 3. Results and Discussion

Chloroplast genomes are well-established as providing variable areas useful for plant identification and studying evolutionary relationships between species. The genetic variations found in chloroplast genome sequencing, such as insertion/deletions (indels) and single nucleotide polymorphisms (SNPs), have been useful for plant identification [21,22]. DNA barcodes of different chloroplast genome regions, such as the *matK* gene, *rbcL* gene, and *trnH*–*psbA* and *trnL-F* spacers, have been successfully utilized for authentication studies [23,24].

In this study, earlier published complete chloroplast genome sequences of lingonberry and American cranberry [20] were utilized for screening variable regions suitable for developing a simple PCR-based method for the authentication of these closely related species. The alignment of the chloroplast genomes led to the identification of several divergent areas suitable for primer design with amplicons less than 200 bp in length, which are suitable for mini-barcoding. Altogether, seven species-specific primer pairs for lingonberry and sixteen for cranberry were designed and tested from the most divergent areas, including the *rbcL–atpB*, *psbZ*–*trnfM*, *trnI-CAU*–*trnL-CAA*, *rps16*–*rrna16*, and *ndhG*–*ndhI* loci. One primer pair for each species from the *trnI-CAU–trnL-CAA* locus, designed by utilizing indel regions, showed high species specificity and high efficiency (Table 1), and these were selected for developing dPCR method. Earlier, Kim et al. (2020) [20] identified the *ycf2-trnL-CAA* locus as one of the most variable intergenic spacer regions in chloroplast genomes among the five studied *Vaccinium* species; however, no clear distinction was then reported between lingonberry and American cranberry in the locus.

**Figure 2:**
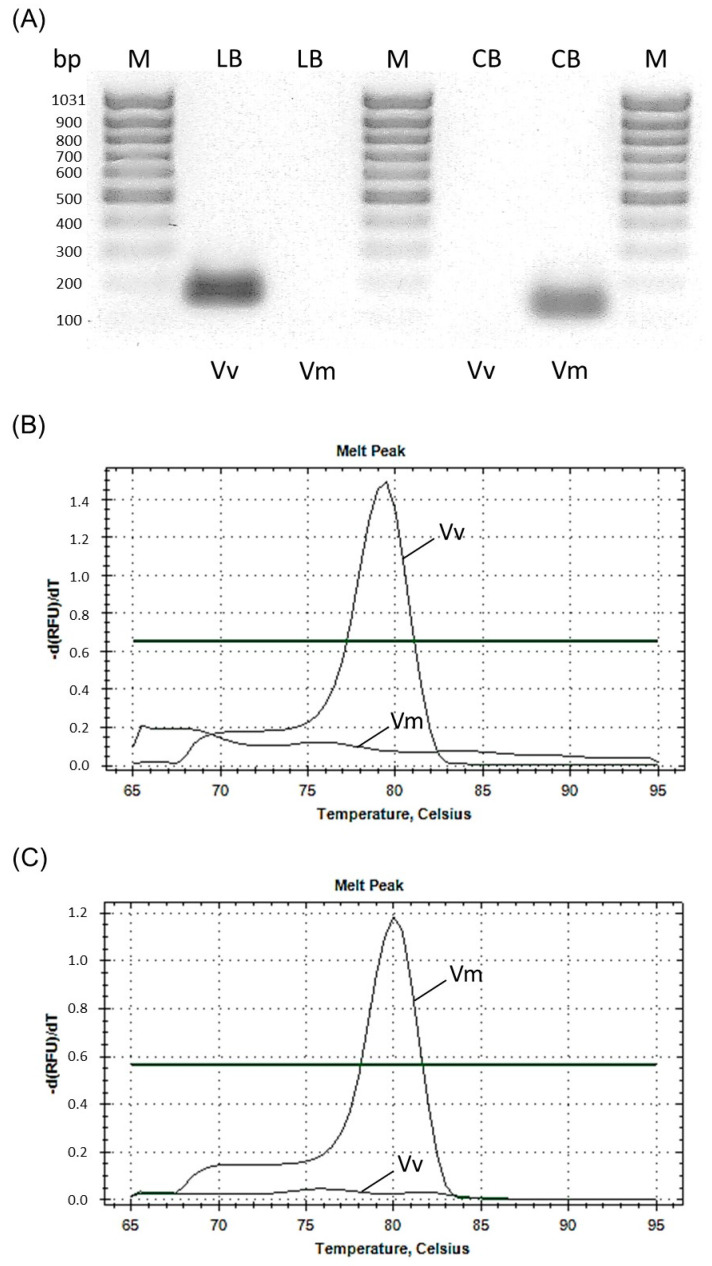
Specificity of the primers by conventional PCR with PCR products shown in 1% agarose gel (**A**) and by qPCR melting curve analysis with lingonberry specific primers (**B**) and American cranberry specific primers (**C**). LB, lingonberry primers; CB American cranberry primers; Vv, *Vaccinium vitis-idaea* DNA; Vm, *Vaccinium macrocarpon* DNA.

The selected species-specific primers were able to amplify only target DNA and thus distinguish between lingonberry and cranberry DNA that was verified by both conventional PCR and qPCR (Figure 2). Conventional PCR amplified only target DNA, with the expected product size being 174 bp for lingonberry and 136 bp for cranberry (Figure 2A, Table 1). The melting curve analyses by qPCR followed by the sequencing of the product further confirmed the amplification of only the target DNA fragment (Figure 2B,C).

Previously, An et al., 2019 [25] developed a qPCR method for identifying four diverse berry fruits (*Aronia melanocarpa*, *Rubus fruticosus*, *V. macrocarpon*, and *Fragaria* x *ananassa*) by using species-specific primers utilizing SNPs and indel regions of *matK*, *trnL-F* and *rbcL* chloroplast gene sequences. They also utilized amplicons under 230 bp in length, which are suitable for mini-barcoding, benefiting from high-speed amplification and have the ability to overcome challenges in detecting partly degraded DNA in processed food products [8,26,27]. Earlier, Wu et al. (2018) [11] successfully used a combination of *rbcL*, *ITS* and *psbA-trnH* mini-barcodes in Sanger sequencing for the authentication of berry products to avoid the influence of processing on DNA amplification. Mini-barcodes have also been used for the authentication of processed medical herbs, green tea, jams, and yogurt [28,29,30,31]. However, in these studies, sequencing was required after PCR to reveal the species identification, while our developed method utilizing the indel regions avoids sequencing enabling a fast analysis time. Our species-specific primers also avoid using additional, expensive fluorescent probes, which are commonly used in qPCR and dPCR based species authentication [15,16,32,33].

Although DNA barcoding by PCR, or in combination with HRM analysis, can be efficiently used in identifying species, the quantification of the species content in mixtures requires the ability to quantify the DNA amount. In this study, we further used the species-specific primers for developing a quantifiable dPCR method for measuring DNA copy numbers in mixed DNA samples. The results show that the method is very sensitive, being able to detect the mixing of even a 1% cranberry DNA sample in a lingonberry DNA sample and vice versa (Table 2). Both primer pairs produced reliable results in different concentrations of the mixed lingonberry/cranberry DNA samples. Our results are in accordance with earlier results. Yu et al. (2021) [19] studied the power of the droplet digital PCR (ddPCR) method for the quantification of *Panax notoginseng* root powder in mixed samples with 10–60% addition of rice, potato or soyabean powders or all three within the same sample. The results showed 0.73–11.65% deviation in accuracy of the ddPCR result in quantification of *P. notoginseng* DNA concentration in the mixed samples. Similarly, a high accuracy with a relative error rate of 0.02–0.43% was shown in dPCR analysis of mixed samples of common wheat (*Triticum aestivum*) and durum wheat (*T. durum*). The assay was developed for detecting common wheat contamination in Italian pasta production using pure durum wheat, in which the threshold for the contamination is set to 3% by Italian laws [34]. For meat products, 0.01% accuracy for the dPCR identification of fraudulent raw materials and products has been reported [14,35].

Compared to qPCR, dPCR avoids the use of standard curves and is more sensitive and accurate in the calculation of the absolute DNA copy numbers [13]. In our study, the dPCR quantification method could be reliably used at least down to the 1% (*w*/*w*) level for lingonberry and American cranberry DNA mixtures. For the berry industry, this accuracy is more than enough because the fraudulent products in this case would potentially be replacing the majority of the lingonberry raw material with American cranberry, and minor additions (<1%) would hardly affect the quality of the product.

## 4. Conclusions

In this study, we have developed a simple, rapid, and quantitative method for the measurement of the absolute copy number of lingonberry and American cranberry DNA by dPCR. The sensitivity of the method is suitable for the needs of the berry industry even without expensive probes since there is no need to show fraud samples under 1% in the case of lingonberry–cranberry mixes. Furthermore, the mini-barcoding method described in this study, could be further developed for the amplification of partly degraded DNA of processed food products. Due to the use of species-specific primers amplifying only target DNA, there is no need for the sequencing of PCR amplicons providing fast authentication analysis. The same method utilizing chloroplast indel regions can be optimized to other related species often mixed into berry products, for instance wild bilberries, which are often mixed with cultivated blueberries (*V. myrtillus* vs. *V. corymbosum* or *V. angustifolium*), but also used for the authentication of other plant species.

## Figures and Tables

**Table 1:** Species-specific primers for *Vaccinium vitis-idaea* and *Vaccinium macrocarpon* from chloroplast genome.

Locus	Locus Location	Primer Sequence 5′-3′	Amplicon Size (bp)	Primer Efficiency (%)
*trnI-CAU–trnL-CAA*	92554–96620 ^1^	LB_F-TAGGCCTTGAAAGGAGAAGGAG	174	104.7
(*Vaccinium vitis-idaea*)		LB_R-GCTCGTAATCCAGCCGATAAAG		
*trnI-CAU–trnL-CAA*	95178–98419 ^2^	CB_F-CGTGCATTAAGACACGAAGG	136	108.6
(*Vaccinium macrocarpon*)		CB_R-TAAGGCTCCACTGCCTATGG		

^1^ In *Vaccinium vitis-idaea* chloroplast genome GenBank accession no. LC521969 [20]. ^2^ In *Vaccinium macrocarpon* chloroplast genome GenBank accession no. NC_019616 [20].

**Table 2:** dPCR identification of lingonberry and cranberry DNA in mixed DNA samples.

	Lingonberry		Cranberry	
DNA Mix (*w*/*w*) %	DNA Copies per µL	Measured Value %	DNA Copies per µL	Measured Value %
100/0	860 ^1^	100	1374	100
99/1	851	98.95 (−0.05)	1322	96.22 (−2.80)
95/5	847	98.49 (+3.67)	1257	91.48 (−3.71)
90/10	828	96.28 (+6.98)	1170	85.15 (−5.39)
75/25	729	84.77 (+13.03)	1003	73.00 (−2.67)
50/50	490	56.98 (+13.96)	682	49.64 (−0.72)
25/75	233	27.09 (+8.36)	347	25.25 (+1.00)
10/90	84	9.77 (−2.30)	153	11.14 (+11.40)
5/95	52	6.05 (+21.00)	66	4.80 (−4.00)
1/99	10	1.16 (+16.00)	12	0.87 (−13.00)
0/100	0	0	0	0

^1^ The results are presented as average of replicates with deviation % in brackets.

## Data Availability

Data is contained within the article.
